# Compatibility between an overnight fasting and random cholesterol tests in Asians

**DOI:** 10.1038/s41598-021-85914-y

**Published:** 2021-03-19

**Authors:** Ian Kwong Yun Phoon, Yi Ling Eileen Koh, Xiaoxuan Guo, Sankari Usha, Ngiap Chuan Tan

**Affiliations:** 1 c/o SingHealth Polyclinics, 167, Jalan Bukit Merah, Connection One, Tower 5, #15-10, Singapore, 150167 Singapore; 2 SingHealth-Duke NUS Family Medicine Academic Clinical Programme, 31 Third Hospital Avenue, # 03-03, Bowyer Block C, Singapore, 168753 Singapore

**Keywords:** Diabetes, Dyslipidaemias

## Abstract

Recent Western guidelines recommend non-fasting lipid profiles to manage dyslipidaemia. We explored its applicability to an Asian population. We determined the differences between an overnight fasting and non-fasting cholesterol profiles of patients with type-2 diabetes mellitus (T2DM) in Singapore. We studied 470 multi-ethnic Asian adult patients with T2DM and dyslipidaemia from 2 primary care clinics in Singapore. Non-fasting blood specimens were collected within 6 h after their last meal and within 14 days of a fasting specimen. The intraclass correlation coefficient (ICC) was used to compare the intraindividual lipid profiles. An ICC value > 0.75 implies good correlation. The mean age and T2DM duration of the study population were 62.5 years and 9.8 years respectively. Their mean non-fasting period was 2.46 h. The mean differences between non-fasting and fasting total cholesterol (TC), high-density lipoprotein (HDL-C), triglyceride (TG), low-density lipoprotein (LDL-C), and non HDL-C were + 0.04 mmol/l, − 0.001 mmol/l, + 0.48 mmol/l, − 0.15 mmol/l, and − 0.05 mmol/l respectively. The ICC (95% CI) for TC, HDL-C, TG, LDL-C and non-HDL-C were 0.820 (0.788–0.847), 0.873 (0.850–0.893), 0.579 (0.516–0.636), 0.764 (0.723–0.799), and 0.825 (0.794–0.852) respectively. The fasting and non-fasting lipid profiles were similar in our local Asian patients with T2DM taking statin. Their non-fasting lipid profile can be used to assess their cholesterol treatment status.

## Introduction

Cardiovascular disease is one of the leading causes of death in non-communicable disease in developed countries^1^. Dyslipidemia and type-2 diabetes mellitus (T2DM) are known risk factors for cardiovascular disease^2^. Reducing low-density lipoprotein cholesterol (LDL-C) level has been shown to be strongly associated with decreased risk of coronary artery disease^3,4^. Assessing the dyslipidemia status conventionally requires individuals to fast for 8–12 h^5^. It creates challenges in operationalizing the phlebotomy services, where appointments will be constrained by the narrow window period in the morning to cater to large groups of fasting persons. The patients may be adversely affected by fasting, such as hypoglycaemia^6^.

Recent studies have also challenged this convention of using fasting cholesterol panels in the management of patients with dyslipidaemia^7–9^. Based on a large cohort study in Copenhagen^9^, the difference of a non-fasting LDL-C with a fasting one was − 0.2 mmol/l, and was deemed not clinically significant. The NHANES III data revealed no difference in risks of all-cause mortality and cardiovascular related mortality when fasting and non-fasting LDL-C was used^10^. The results of the post hoc Anglo-Scandinavian Cardiac Outcome Trial-Lipid Lowering Arm (ASCOT LLA), showed a high concordance (94.8%) of fasting and non-fasting lipid levels for classifying participants into ASCVD risk categories^11^. In addition, the recent American Heart Association (AHA) and the European Society of Cardiology (ESC) guidelines have suggested that fasting lipid profiles are no longer required for the monitoring of dyslipidemia^12,13^.

Nonetheless, the evidence are based largely on Caucasian studies^7–11^. Asians have different cardiovascular risk profiles from the Caucasians, where stroke is more prominent as compared to coronary heart disease (CHD)^14^. South Asians (e.g. Indians) are also thought to have an excess risk for CHD beyond traditional risk factors^15^. Furthermore, the World Health Organisation (WHO) experts recommend a lower body-mass index (BMI) for Asians as compared to the West^16^, reflecting that extrapolation of data from the West to Asia is not a given. Asia is now the global epicentre for the surge in prevalence of type-2 diabetes mellitus^17^ in many developing and developed nations. Evaluation of their risk factors, including their lipid profiles becomes critical in clinical intervention to mitigate their cardiovascular risks. The distinction between fasting and non-fasting LDL-C becomes compelling in the cardiovascular risk assessment of Asians.

Singapore is a microcosm of multi-ethnic Asians at the centre of South-east Asia, with a population of 5.7 million^18^, comprising largely of Chinese, Malays and Indians^19^. The local government primary care centres (called “polyclinics”) are the ideal sites to assess if there is significant difference between fasting and non-fasting lipid profiles in these three large Asian ethnic groups.

As insulin resistance in T2DM can influence the lipid profile significantly by raising the triglyceride (TG) and lowering the high density lipoprotein cholesterol (HDL-C)^20,21^ we focused on patients with a history of T2DM and dyslipidaemia on statin treatment. We postulated that the difference between the non-fasted and fasted lipid profile would show excellent inter-class correlation (ICC), thus making the non-fasting lipid profile suitable for the monitoring and treatment of dyslipidaemia in Asians.

## Method

### Study sites

The study sites were 2 polyclinics located in Pasir Ris and Punggol estates in north-eastern Singapore. These 2 polyclinics serve a population of 318,580 (June 2019) comprising 71.6% Chinese, 17.2% Malay and 7.9% Indians^22^, with daily attendances of 600–900 patients on each weekday.

### Study population

A prospective intraindividual comparison study was conducted on adult patients, aged 21 years and above, with diagnoses of both T2DM and dyslipidaemia on statin therapy based on their ICD-10 disease codes in their electronic medical records (EMR). They had two lipid profiles: one overnight fasting and another non-fasting blood specimen collected within 14 days apart. Fasting is defined as without any food consumption 8 or more hours before the blood test. Intake of plain water was allowed. Non-fasting is defined as the last meal or beverage of less than 8 h before the blood test. The patients were currently on statin therapy of any brand, with no changes in dose, frequency or type of statin or any other lipid lowering drug for 12 months or more prior to recruitment, based on their prescription records in the EMR.

The following exclusion criteria applied to the study population:

- non-residents
- patients who were pregnant,
- patients with record of fasting triglyceride level of ≥ 4.5 mmol/l

Recruitment began in March 2018, and ended in December 2019.

### Recruitment of patients

Potential patients were screened by doctors in the 2 primary care centres when they came for their routine visit, usually 1 week after they have done a fasting lipid profile. A brief description of the study was explained to the patient, and if interested, they would be sent to a research assistant, whereby a more detailed screening is done to ensure eligibility, before informed consent was obtained. To encourage participation, a Singapore $10 grocery voucher was offered for all participants who had completed the questionnaire and the non-fasting lipid profile.

### Demographic and laboratory data

The patient’s age, gender and race, medical history, HbA1c, current medications, details of meals, beverage and blood sampling were documented in a short questionnaire. Time from the end of the last meal to blood taking was computed for each patient and rounded down to the nearest hour.

The blood samples were sent from the polyclinic laboratories to the central Pathological Laboratory in the Singapore General Hospital twice daily. The specimens were processed using the Roche Cobas c702 module, which leveraged on spectrophotometry to measure the TC, HDL-C, and TG. The LDL-C was calculated based on the Friedewald formula, so no value would be obtained if the TG was > 4.5 mmol/l. The Friedewald formula is:

$$LDL\text{-}C = Total Cholesterol levels\; ({TC}) - HDL\text{-}C - \left[ {TG/2.2} \right]{\text{mmol/l}}\;\left( {or\;\left[ {TG/5\,{\text{mg/dl}}} \right]} \right)$$

and only when TG is < 4.5 mmol/l (TG < 400 mg/dl), beyond which the formula is deemed inaccurate^23^. The results were reported daily and automatically channelled to the EMR, which were then retrieved by the research coordinators and recorded in the study documents. The Systeme International (SI) units “mmol/l” was used to define each component of the lipid profile (1 mmol/l cholesterol = 38.7 mg/dl).

### Data management

All subject’s data were kept in a secure database (password protected) for analysis. Patient identifiers were removed, and each subject assigned a study number by the research assistant. Hard copy data collected were updated into the computerised database by these research assistants. The completeness and integrity of the data was checked by one of the authors, Ms Sankari Usha, who oversaw data management.

### Statistical analysis

Our study objective is to compare the agreement of two continuous measurements, fasting and non-fasting lipids of the same individual, using the intra-class correlation (ICC). Each person underwent two measurements: fasting and non-fasting lipid profile. We estimated that the ICC to be within ± 0.025, 95% CI width = 0.05. If we assume the planed ICC = 0.85, according to the sample size tables for clinical studies, the required sample is 475 subjects, and the expected 95% CI ranges from 0.825 to 0.875. We choose to recruit 476 subjects.

Each patient’s fasting lipid profile and non-fasting lipid profile was compared. The mean and standard deviation of the differences in TC, HDL-C, TG, calculated LDL-C, and Non-HDL-C between the fasted and non-fasted states of each individual were presented. The agreement between the two readings were compared using two-way mixed effects intra-class correlation coefficient (ICC). An ICC > 0.9 indicates excellent reliability, 0.75–0.9 good reliability, 0.5–0.75 fair reliability, and ICC < 0.5 indicates poor reliability^24^. Differences for the non-fasting and fasting TG in the various food items were assessed using independent t-test. All analyses were performed using IBM SPSS version 25.0 software.

In addition we performed a Medication Adherence Report Scale (MARS-5) with regards to the patients’ adherence to statin treatment. MARS-5 is a 5-question questionnaire that was developed and validated by Professor Rob Horne to measure patients’ adherence to treatment^25^. Each question pertaining to how the patient adheres to the prescribed instructions is scored from 1 to 5 (1 = always, 2 = often, 3 = sometimes, 4 = rarely, 5 = never), with the total score ranging from 5 to 25. A high score (close or equal to 25) would indicate good adherence.

### Study, ethics approval and funding

The study protocol and ethics were reviewed, approved and audited by SingHealth’s Centralised Institutional Review Board (CIRB, Number 2018/2167). Informed consent was obtained for all subjects, and we complied with all the institution’s ethical guidelines. The study was funded by the SingHealth Polyclinics Research Support Programme.

## Results

**Figure 1:**
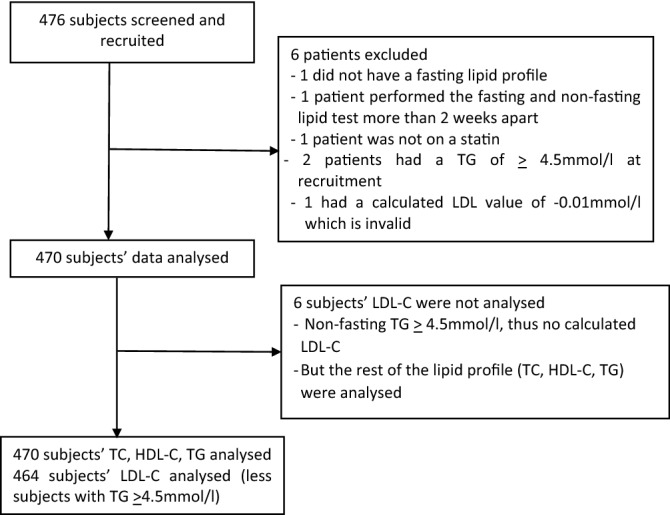
Consort flow diagram.

We recruited a total of 476 subjects and analysed the data for 470. A summary of this recruitment is shown in Fig. 1. Six out of 470 subjects (1.28%) had non-fasting TG > 4.5 mmol/l (> 400 mg/dl), and did not have a calculated LDL-C, and thus were excluded in the ICC analysis between the fasting and non-fasting LDL-C.

Table 1 shows the demographics of the 470 subjects. The racial proportion is close to that of Singapore’s national racial mix^19,22^, with slightly more Malays, and less Chinese: Chinese (60.6%), Malays (27.5%), and Indians (8.5%) being the 3 largest racial groups, and with 3.4% being Eurasians. 51.7% of the subjects were male. Mean age was 62.5 (± 9.1) years, and mean duration of diabetes is 9.8 (± 8.7) years. 58 (12.3%) were on insulin. Mean HbA1c was 6.7% (± 0.8) [49.7 mmol/mol (± 0.86)].

**Table 1:** Study population demographics (n = 470) distribution of statin type and intensity, and MARS-5 score distribution for adherence to statin treatment.

	Frequency (%)
Total	470 (100.0)
Age (years), mean (SD)	62.5 (9.1)
**Gender**	
Male	243 (51.7)
Female	227 (48.3)
**Ethnic group**	
Chinese	284 (60.4)
Malay	130 (27.7)
Indian	40 (8.5)
Eurasian/others	16 (3.4)
Duration of DM (years), mean (SD)	9.9 (8.7)
**Clinical parameters**	
Weight (kg), mean (SD)	70.2 (14.1)
Height (m), mean (SD)	1.6 (0.1)
BMI (kg/m^2^), mean (SD)	27 (4.5)
Last HbA1c (%), Mean (SD)	6.7 (0.8)
**Low intensity statin**	
Lovastatin 5–40 mg	34 (7.2)
Simvastatin 5–20 mg	160 (34.1)
Atorvastatin 5–10 mg	32 (6.8)
**Moderate intensity statin**	
Simvastatin 30–40 mg	51 (10.9)
Atorvastatin 20–30 mg	66 (14.1)
Rosuvastatin 5 mg	2 (0.4)
**High intensity statin**	
Atorvastatin 40–80 mg	113 (24.1)
Rosuvastatin 10–40 mg	10 (2.1)
**MARS-5 Questions**	Mean score (SD)
I forget to take my Statin	4.8 (0.6)
I change the dosage of my Statin	4.8 (0.6)
I stop taking my Statin for a while	4.9 (0.4)
I decide to skip one of my Statin dosages	4.9 (0.5)
I use my Statin less than is prescribed	4.9 (0.4)
**MARS-5 score**	
Complete adherent (MARS5 = 25)	305 (64.9)
Incomplete adherence (< 25)	165 (35.1)

Table 1 also shows the type and dose of statin the patients were on. 226 (48.0%) were on low dose statin (equivalent to atorvastatin 10 mg or less a day), 120 (25.4%) were on moderate dose statin (equivalent to atorvastatin 20–30 mg a day), and 123 (26.1%) were on high dose statin (equivalent to atorvastatin 40 mg a day or more). Mean time between the last meal and non-fasting blood test was 2.46 (0.5–6) hours.

Table 1 (bottom) also shows the MARS-5 score for statin adherence. All 470 patients completed the questionnaire. Adherence was very high, with 64.9% getting the full score of 25, with all questions averaging between 4.8 to 4.9 out of 5.

Mean difference between non-fasting and fasting TC, HDL-C, TG, LDL-C, and Non HDL-C were + 0.04 mmol/l, − 0.01 mmol/l, + 0.48 mmol/l, − 0.15 mmol/l, and − 0.05 mmol/l respectively (Table 2). Interestingly, all the values were statistically different, using the paired t-test, except for HDL-C.

**Table 2:** Difference of fasting and non-fasting lipid readings using paired t-test.

Lipid/mmol/l	Fasting (SD)	Non-fasting (SD)	Mean difference (95% CI)	*P* value
TC	3.97 (0.76)	4.01 (0.79)	+ 0.043 (+ 0.001 to + 0.085)	0.045
HDL-C	1.32 (0.32)	1.31 (0.34)	− 0.011 (− 0.026 to + 0.004)	0.156
TG	1.42 (0.59)	1.90 (0.87)	+ 0.484 (+ 0.422 to + 0.545)	< 0.001
LDL-C	1.99 (0.63)	1.84 (0.65)	− 0.154 (− 0.194 to − 0.114)	< 0.001
Non-HDL-C	2.65 (0.73)	2.70 (0.78)	+ 0.054 (+ 0.014 to + 0.095)	0.009

Intra-class correlation (ICC) (95% CI) was 0.820 (0.788–0.847), 0.873 (0.850–0.893), 0.579 (0.516–0.636), 0.764 (0.723–0.799), and 0.825 (0.794–0.852) respectively (Table 3). ICC values ≥ 0.75 indicate good correlation^24^. All components except TG achieved this. TG however, achieved moderate co-relation (ICC from 0.5 to 0.75).

**Table 3:** Intraclass correlation (ICC) agreement of fasting and non-fasting lipids. ICC value ≥ 0.75 indicates good corelation^24^.

ICC pairing	ICC (95% CI)	ICC Correlation
Fasting and non-fasting TC	0.82 (0.79–0.85)	Good
Fasting and non-fasting HDL-C	0.87 (0.85–0.89)	Good
Fasting and non-fasting TG	0.58 (0.52–0.64)	Moderate
Fasting and non-fasting LDL-C	0.76 (0.72–0.80)	Good
Fasting and non-fasting non-HDL-C	0.83 (0.79–0.85)	Good

Table 4 shows some the types of food and/or drinks taken. Bread, buns, biscuits or crackers (carbohydrate-based foods) was the most common food taken (44.7%), and coffee (50.6%) and other milk based beverages (32.2%) are the most common drinks. Table 5 shows that it did not matter what they consumed. All the TG was significantly higher when not fasted.

**Table 4:** Distribution of types of food and drinks taken before the non-fasting lipid test as declared by the subjects.

What did you eat?	Frequency (%)
Nothing	3 (0.6)
Bread/buns/biscuits/crackers	211 (44.9)
Rice/porridge/potatoes/carrot cake	100 (21.3)
Noodles/Kway Teow (form of noodle)	114 (24.3)
Cakes/chocolate/ice-cream/cheese/butter/yogurt	23 (4.9)
Roti prata/curry puff/Kueh/You tiao (snacks)	39 (8.3)
Meats/eggs/bean curd	186 (39.6)
Fruits/vegetables	119 (25.3)
Others	150 (31.9)

What did you drink	
Nothing	11 (2.3)
Water	145 (30.9)
Milk/creamer/condensed or evaporated milk/milo/Horlicks/Ovaltine/cocoa (beverages)	152 (32.3)
Coffee	239 (50.9)
Tea (including Chinese, green tea, exclude bubble tea)	81 (17.2)
Fruit juice	4 (0.9)
Soft drinks, sweetened drinks, bubble tea	8 (1.7)
Soyabean milk	4 (0.9)
Soup	5 (1.1)
Others	48 (10.2)

**Table 5:** Association of non-fasting TG and food items consumed using independent t-test.

	Non-fasting TG/mmol/l	Fasting TG/mmol/l	*P* value
Carbohydrate	1.91 (0.88)	1.42 (0.6)	< 0.001
Meat	2 (0.9)	1.46 (0.53)	< 0.001
Vegetables	2.02 (0.87)	1.36 (0.49)	< 0.001
Milk-based beverages	1.84 (0.76)	1.37 (0.55)	< 0.001
Coffee/tea	1.9 (0.86)	1.39 (0.61)	< 0.001
Fruit juice and sweetened beverages	2.41 (1.51)	1.6 (0.8)	0.068
Soup	1.5 (0.53)	1.52 (0.84)	0.602

Carbohydrate includes: bread/buns/biscuits/crackers, rice/porridge/potatoes/carrot cake, noodles/Kway Teow, cakes/chocolate/ice-cream/cheese/butter/yogurt, roti prata/curry puff/Kueh/You tiao.

Milk based beverages includes: milk/creamer/condensed or evaporated milk/milo/Horlicks/Ovaltine/cocoa.

## Discussion

Our study found that, in a multi-ethnic Asian population with T2DM and dyslipidemia on stable statin dose, the lipid profile changed little after a typical meal. Despite the paired t-test showing significant differences in all lipid components except the HDL-C, the absolute difference other than TG was very small, ranging from − 0.15 to + 0.05 mmol/l. Other than TG, the ICC showed good co-relation between the non-fasting and fasting lipid values, whist TG showed moderate correlation.

Our outcomes were remarkably similar to the Copenhagen Study^9^ on a Danish population, with the mean LDL-C difference even smaller (− 0.15 mmol/l vs − 0.2 mmol/l respectively), despite the greater rise in TG (+ 0.48 mmol/l vs + 0.3 mmol/l respectively). All patients in our study had T2DM (as compared to the Danish study), where we expect a higher level of TG^20,21^. The average time of the last meal was 2.46 h, which was close to the 4 h duration in which the TG was observed to reach post prandial peak^26^. In addition, our Asian diet may contain more fat than a typical Danish diet. In this study, almost one-third (32.2%) of the patients had a milk-based beverage before the non-fasting blood test. All food and beverage categories were associated with significant rise in TG (Table 5), so it does not matter significantly what they ate or drank.

There was high adherence to statin treatment, based on the MARS-5 scores. 64.9% of patients scored a full 25 points. This makes it unlikely that there would be significant variations in therapy between the 2 lipid tests.

In most laboratories, including the SingHealth laboratories, LDL-C is calculated from the Friedewald formula, rather than a direct measurement. A Copenhagen study successfully validated the use of the Friedewald formula to calculate LDL-C using non-fasting blood samples^9^. Furthermore, the authors found that the difference between a non-fasting (< 8 h since the last meal) lipid, as compared to a fasting one was TC − 0.2 mmol/l, HDL-C − 0.1 mmol/l; TG + 0.3 mmol/l; LDL-C − 0.2 mmol/l, which were deemed to be clinically insignificant. In fact, the non-fasting LDL-C appeared to be lower than the fasting ones, which the authors hypothesize could be due to dilution from hydration. In this study, we did not restrict patients from their water intake prior to their fasting blood sampling. We however did not assess the patients’ hydration status with serum albumin or osmolarity in this study. This may be considered in future studies.

An examination of the Friedewald formula would suggest that, assuming TC and HDL-C changes little with food, then the calculated LDL-C would fall as TG rises after a meal^27^. This would mean that if a random LDL-C is elevated, it will truly be elevated, perhaps even more so, in the fasting specimen. Thus, in this scenario, the clinical decision to intensify statin treatment would not be changed by the non-fasting state.

Dyslipidaemia management in Singapore is guided by LDL-C thresholds (or targets)^28^. In view of the insignificant difference between the fasting and non-fasting LDL-C result, treatment decision to titrate statin dose is unlikely to change if random lipids were used, thus not changing clinical practice. With 73.4% of our subjects on low to moderate intensity statins, there is room to titrate up if this is necessary.

However, the impact on the patients and the laboratory services will be significant if random lipids were systematised. The morning wait time and congestion at the laboratory will be reduced as patients can be scheduled to use its service throughout its operational hours. Their discomfort, inconvenience and potential adverse effects of fasting can also be mitigated. The laboratory staff will no longer need to verify if patients have fasted, thus further increasing their efficiency. The implementation and cost-effectiveness of introducing non-fasting lipid panels routinely in polyclinics need to be further evaluated in future study.

## Strength and limitations of the study

### Strengths

The evaluation of the fasting and non-fasting lipid profiles within the same individual prospectively within 14 days apart, with no changes to their statin dose underpins the strength of the study. This mitigates any potential variations which may be attributed to differences in the metabolism due to different age, race or gender. The short time interval, and the absence of any study intervention nor medication changes in between the two blood samples, would suggests that the lifestyle and other potential confounders of each patient are unlikely to significantly alter their lipid metabolism. The MARS-5 scores also showed high adherence to statin treatment, making it less likely that there was a difference in treatment effect between the 2 lipid tests.

Moreover, we focused on patients with T2DM, a high-cardiovascular risk group, as insulin-resistance can significantly affect the lipid profile by raising the TG and lowering the HDL^20,21^.

### Limitations

We did not use direct LDL-C measurements to verify the actual levels of LDL-C in the fasted and non-fasted samples. The current laboratory report of LDL-C relies on its auto-computation using the Friedewald formula. The intent was to conduct the study in a real-world setting, so that the findings can be seamlessly transited from research to implementation in clinical practice without major adjustment to the healthcare system. In addition, we did not study any other lipid particles like VLDL, or chylomicrons as these are not routinely done in our laboratories, and are expensive to do.

The data on the food and drinks that the patient took before the non-fasting lipid test was purely based on the patients’ best recollection. So detailed analysis of their food intake was not possible other than to put then into broad groups.

The study population was 6 short of the 476 subjects stipulated in the sample size computation. This should not have changed the results of the findings significantly. Of the 470 subjects with fasting TG < 4.5 mmol/l at recruitment, only 6 had a non-fasting TG of ≥ 4.5 mmol/l (Fig. 1). These patients would need fasting lipid profile subsequently to determine their LDL-C status. Alternatively, they would have to undertake the direct LDL-C measurement in a separate laboratory, which is not feasible in this study. Nevertheless, only 1 in 78 subjects (6 out of 470) did not have an LDL-C result in their non-fasting lipid profile, as a result of the high TG. This small number is unlikely to significantly affect the conclusion of the study.

The study population was not stratified by ethnicity, as the power of the study is inadequate to carry out any meaningful sub-group analysis to see if different ethnic groups showed a different performance between their non-fasting and fasting lipids. A larger study, with more ethnic minorities may help to clarify this.

## Conclusion

The results demonstrated a good intra-class correlation (ICC) between a non-fasted and fasted lipid profile in Asian adult in Singapore with T2DM and dyslipidaemia on a statin, except for a rise in TG. The mean difference in TG and LDL-C respectively was + 0.48 mmol/l and − 0.15 mmol/l respectively. The small difference supports the latest American and European guidelines^12,13^ that non-fasting lipid profile can be used in the management and monitoring of patients with dyslipidaemia in Asians in Singapore with T2DM on statin treatment.

## Data Availability

The raw data for this study is available upon request to the corresponding author.
